# Growth of Pseudomorphic GeSn at Low Pressure with Sn Composition of 16.7%

**DOI:** 10.3390/ma14247637

**Published:** 2021-12-11

**Authors:** Joshua Grant, Grey Abernathy, Oluwatobi Olorunsola, Solomon Ojo, Sylvester Amoah, Emmanuel Wanglia, Samir K. Saha, Abbas Sabbar, Wei Du, Murtadha Alher, Bao-Hua Li, Shui-Qing Yu

**Affiliations:** 1 Department of Electrical Engineering, University of Arkansas, Fayetteville, AR 72701, USA; jmg029@uark.edu (J.G.); gabernat@uark.edu (G.A.); ogolorun@uark.edu (O.O.); soojo@uark.edu (S.O.); samoah@email.uark.edu (S.A.); eswangil@uark.edu (E.W.); afsabbar@uark.edu (A.S.); maalher@uark.edu (M.A.); 2 Microelectronics-Photonics Program, University of Arkansas, Fayetteville, AR 72701, USA; 3 Department of Physics, University of Arkansas, Fayetteville, AR 72701, USA; sksaha@uark.edu; 4 Department of Electrical Engineering and Physics, Wilkes University, Wilkes-Barre, PA 18766, USA; wei.du@wilkes.edu; 5 Mechanical Engineering Department, University of Kerbala, Kerbala 56001, Iraq; 6 Arktonics, LLC, 1339 South Pinnacle Drive, Fayetteville, AR 72701, USA; arktonics@gmail.com; 7 Institute for Nanoscience and Engineering, University of Arkansas, Fayetteville, AR 72701, USA

**Keywords:** group-IV, GeSn, CVD growth, low pressure

## Abstract

Group-IV alloy GeSn holds great promise for the high-performance optoelectronic devices that can be monolithically integrated on Si for near- and mid-infrared applications. Growth of GeSn using chemical vapor deposition technique with various Sn and Ge precursors has been investigated worldwide. To achieve relatively high Sn incorporation, the use of higher pressure and/or higher order Ge hydrides precursors were reported. In this work, we successfully demonstrated the growth of high-quality GeSn with Sn composition of 16.7% at low pressure of 12 Torr. The alloy was grown using the commercially available GeH_4_ and SnCl_4_ precursors via a chemical vapor deposition reactor. Material and optical characterizations were performed to confirm the Sn incorporation and to study the optical properties. The demonstrated growth results reveal a low-pressure growth window to achieve high-quality and high Sn alloys for future device applications.

## 1. Introduction

GeSn alloys offer promising optical advantages compared to traditional group-IV semiconductor materials such as Si and Ge [1,2,3]. With sufficiently high Sn composition, the true direct bandgap GeSn has led to the successful demonstration of optically pumped and electrically injected GeSn lasers [4,5,6,7,8]. The tunable bandgap covering broad near- and mid-infrared wavelength enables the development of light emitter and detectors towards Si-based longwave integrated optoelectronics (LIO) applications [9,10,11,12]. Moreover, the complementary metal–oxide–semiconductor (CMOS) process compatibility allows GeSn-based devices to be monolithically integrated on Si substrates [13,14,15].

Growth of GeSn alloys on Si substrate is difficult because of the low solubility (<1%) of Sn in Ge, the instability of α-Sn above 13°C, and the large lattice mismatch between GeSn and Si (>4.2%). To address these challenges, non-equilibrium condition growth techniques were developed. Low temperature growth using molecular-beam epitaxy (MBE) [16,17,18] and chemical vapor deposition (CVD) [19,20,21] have been reported. For the past decade, the CVD growth technique has been increasingly investigated worldwide. The early growth utilized deuterium-stabilized stannane (SnD_4_) as the Sn precursor [22,23], which suffered from high-cost and instability, and motivated the use of low cost, stable, and commercially available tin-tetrachloride (SnCl_4_). It has been reported that the SnCl_4_-based growth achieves device-level material quality [20,24]. Additionally, using low cost and commercially available germane (GeH_4_) as Ge precursor was widely reported [25,26,27,28].

From the device application perspective, higher Sn incorporation is desired due to: (i) more bandgap directness enhances the efficiency of emitter; and (ii) an extended spectral response cutoff is in favor of long wavelength detection. To achieve higher Sn composition, higher order Ge hydrides, such as Ge_2_H_6_ and Ge_3_H_8_, were adopted due to their favorable decomposition at low temperature [29,30,31,32,33]. Alternatively, plasma-enhanced CVD growth was successfully demonstrated in an ultra-high vacuum (UHV) chamber [34]. Currently, the Sn incorporation is a figure of merit from the material growth perspective. The summary of GeSn CVD growth results using SnCl_4_ as Sn precursor is shown in Table 1.

It is generally acknowledged that using higher order Ge hydrides precursors would facilitate Sn incorporation. From Table 1, it can be seen that most higher Sn growth work (>10.0%) were accomplished by using Ge_2_H_6_. Moreover, the chamber pressure plays an important role for Sn incorporation, as generally the high Sn materials are grown under higher pressure. The maximum Sn composition of 18.0% was achieved under relatively high pressure of 120 Torr [26]. Despite employing Ge_2_H_6_ or Ge_3_H_8_ offers a viable solution for high Sn material growth, using GeH_4_ is preferred in manufacturing due to its much lower cost; it is worth noting that higher Sn alloys may suffer from deteriorated material quality, limiting the device performance using such material. Therefore, a growth method that meets utilizing industry-preferred precursors, higher Sn incorporation, and device-level material quality is highly desired, which has yet been fully explored.

In this work, we demonstrate the growth of a GeSn alloy with Sn composition of 16.7%. The material was grown using commercially available GeH_4_ and SnCl_4_ precursors via a home-built UHV-CVD reactor. The pressure was kept as low as 12 Torr. Material and optical characterizations were conducted to confirm the Sn incorporation as well as to show optical properties. In addition, the surface defects were analyzed to speculate the growth dynamic. While the in-depth understanding of low-pressure growth is still undergoing, the grown GeSn alloy presented in this work indicates a new growth window towards high Sn and high material quality for future device applications.

## 2. Experimental Methods

The GeSn sample was grown on a 100-mm p-type silicon (100) substrate with the resistivity of 10–20 Ω·cm via a custom built cold walled UHV-CVD reactor. The base pressure of the reactor is 10^−10^ Torr. The silicon substrate was cleaned using a piranha etch solution (H_2_SO_4_:H_2_O_2_ = 1:1), followed by oxide removal and hydrogen passivation solution using the diluted HF. Prior to GeSn film growth, a Ge buffer layer was grown at 1 Torr using a two-step process: the first step growth was performed at 375 °C while the second step was at 600 °C. The flow rate of Ge precursor (GeH_4_) during the second step of the buffer growth was reduced by half of that in the first step. The growth rate is 0.2 µm/min and the overall thickness is 6.6 µm. The GeSn layer growth was performed at 260 °C using the GeH_4_ and SnCl_4_ as Ge and Sn precursors, respectively, with the argon (Ar) acted as the carrier gas. The growth rate was estimated as ~1.33 nm/min.

Material characterizations were performed following the growth. Atomic force microscopy (AFM) was used to investigate the surface morphology. The high-resolution X-ray diffraction (XRD) 2θ-ω scan along the Si (004) plane and the reciprocal space map (RSM) along $\bar{2}\bar{2}4$ were employed to identify the Sn composition, the degree of strain, and the layer thickness. The transmission electron microscopy (TEM) image was obtained to cross-check the layer thickness, as well as to check the material quality.

Optical characterizations were performed to further study the GeSn thin film properties. A Raman spectroscopy setup including a 632 nm HeNe laser pump source by Thor Labs (Newton, NJ, USA) at 5 mW power and a Horiba iHR 550 (Kisshoin, Minami-ku, Kyoto, Japan) grating-based spectrometer with a charge coupled device (CCD) detector was employed to quantify the shift of longitudinal optical (LO) phonon peak. A Ge bulk sample was used as reference. The absorption coefficient spectrum was measured via a J. A. Woollam V-Vase ellipsometer (Lincoln, NE, USA) in the range of 0.496 to 4.768 eV (260 to 2500 nm) with a resolution of 10 nm at incidence angle of 70°. The absorption coefficient was obtained by using the Johs-Herzinger model, and then was fitted by applied physical model [35]. The photoluminescence (PL) study was conducted using the standard off-axis setup configuration and lock-in techniques. A 1064 nm pulsed laser was used as the pumping source. The emissions were collected using a Horiba iHR 320 (Kisshoin, Minami-ku, Kyoto, Japan) grating based spectrometer equipped with a PbS detector with a spectral cutoff at 3.0 µm.

## 3. Results and Discussion

**Figure 1:**
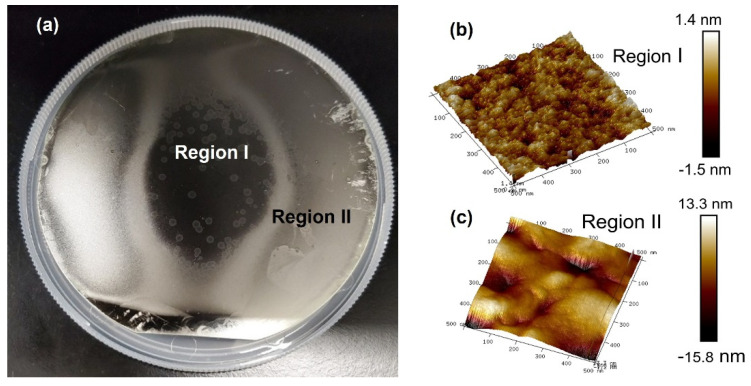
(**a**) Optical image of as grown sample with two distinct regions (Region I: shiny center, and region II: cloudy outer ring). AFM images of (**b**) region I and (**c**) region II showing the surface roughness of ~3 nm and ~30 nm, respectively, indicating a better quality of region I.

Figure 1a shows the optical image of the entire wafer that was taken immediately after the growth was completed. Two distinct regions were observed: a shiny region with some island-like areas at the center (region I), surrounded by a cloudy region (outer ring, region II). The clearly-resolved regions suggest different material quality. Generally speaking, the shiny region indicates a higher quality which will be confirmed via the following material characterizations. Note that there is a “transition” area between the regions I and II, showing a bit different color. As this area features similar surface roughness and morphology after characterization, it was included in region II.

AFM images of 0.5 µm × 0.5 µm area are shown in Figure 1b,c for regions I and II, respectively. Region I features surface roughness of ~3 nm (−1.5 to 1.4 nm) compared to that of region II of ~30 nm (−15.8 to 13.3 nm). The smaller surface roughness indicates a better material quality in region I. The rough surface in region II may be due to the Sn segregation, which was observed in previous study [36].

**Figure 2:**
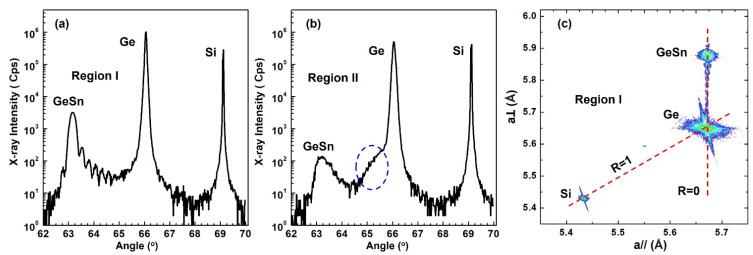
(**a**,**b**) XRD 2θ-ω scans of GeSn film on Ge-buffered Si (100) of region I and II. (**c**) RSM contour plot of region I showing the pseudomorphic growth.

High-resolution XRD was used to examine crystalline characteristics of the sample. Figure 2a shows the 2θ-ω scan of Region I. The well-resolved peaks at 69.1° and 66.1° correspond to Si substrate and Ge buffer, respectively. Based on previous study, incorporation of Sn would shift the peak towards lower angle. Therefore, the peak at 63.1° is assigned to GeSn thin film. The multiple oscillations between 63° and 65° are Pendellösung fringes, which indicates uniformed Sn incorporation and a smooth interface between GeSn and Ge buffer. Moreover, via XRD simulation, the Sn composition and film thickness were fitted as 16.7% and 40 nm, respectively.

The 2θ-ω scan of Region II is shown in Figure 2b. The Si substrate and Ge buffer peaks are the same as in Figure 2a. For the GeSn film, the peak position at ~63.3° is a slightly higher than that in (a). However, the peak linewidth is much broader, indicating the lower material quality of region II compared to region I. The dramatically reduced signal intensity (1.5 order of magnitudes) and the disappearance of Pendellösung fringes also suggest the lower material quality. In addition, two distinct features can be observed in Figure 2b: (i) asymmetric GeSn peak with smaller slope at higher angle side; and (ii) A clear shoulder at ~65° implying the possible existence of a GeSn peak close to Ge reference peak. This can be interpreted as the Sn segregation near the surface, which decreases the Sn incorporation down to less than 3% locally, resulting in an additional GeSn layer with lower Sn composition. At current stage, it is very difficult to accurately position this GeSn layer. Due to the Sn segregation, the Sn composition in region II is a little lower than that in region I.

Figure 2c shows the RSM contour plot of region I. The in-plane (*a*//) and out-of-plane (*a*⊥) lattice constants were extracted from XRD simulation. “R = 1” represents relaxation line and “R=0” indicates the pseudomorphic growth line. It is clear that the Ge buffer is almost strain relaxed while the GeSn thin film is lattice-matched to Ge buffer. By using the Vegard’s law: $a_{Ge_{1-x}Sn_{x}}=\left(1-x\right)a_{Ge}+xa_{Sn}$, where lattice constants for Ge and Sn are 5.646 and 6.489 Å, respectively, the degree of strain can be calculated. The Ge buffer is under a slight tensile strain of 0.17%, and the GeSn film experiences compressive strain of 2.03%.

**Figure 3:**
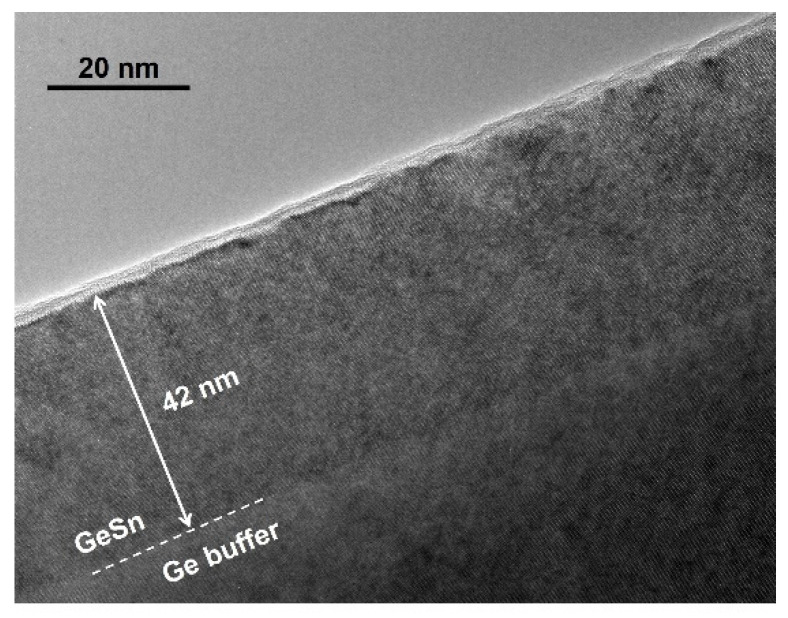
Cross-sectional TEM image showing the GeSn layer thickness of 42 nm.

The sample was further characterized using TEM technique. The cross-sectional image of region I is shown in Figure 3. The interface between GeSn layer and Ge buffer can be clearly resolved (dashed line), where the relatively low density of threading dislocations was observed. Most threading dislocations were localized at GeSn/Ge interface and did not propagate to GeSn layer, resulting in high material quality. The thickness of GeSn was measured as 42 nm, matching with the result obtained from XRD.

**Figure 4:**
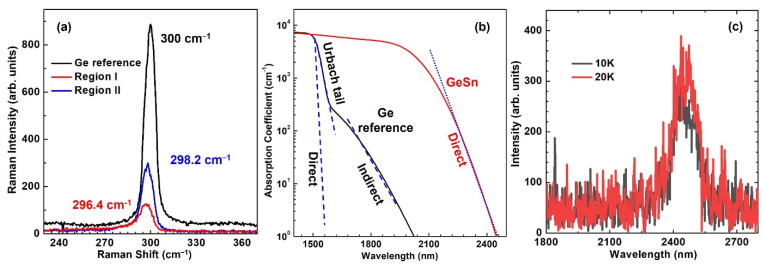
(**a**) Raman spectroscopy showing the shift of Ge-Ge LO phonon peak. (**b**) Spectral absorption coefficient measured using ellipsometry. (**c**) PL spectra at 10 and 20 K.

The optical properties of GeSn thin film were further studied. Figure 4a shows the Raman spectroscopy of the two regions. A Ge bulk sample was used to calibrate the measurement setup, whose Ge-Ge LO phonon peak locates at 300 cm^−1^. For GeSn thin film, the Ge-Ge LO peaks were obtained at 296.4 and 298.2 cm^−1^ for regions I and II, respectively. The peak shift towards lower wavenumber is due to the induced strain by incorporation of Sn atoms into Ge lattice. The more Sn incorporated; the more peak shift can be observed. Therefore, region I features a slightly higher Sn incorporation than region II, which matches the XRD 2θ-ω scan measurement results shown in Figure 2.

The spectral absorption coefficient of region I was measured using ellipsometry spectroscopy, as shown in Figure 4b. The absorption curve of Ge was also plotted as a reference. Compared to Ge, the measured absorption curve shows significant red-shift, indicating the origin of absorption is from GeSn layer. For Ge, since the energy separation between Γ and L valleys is ~140 meV, the absorption associated with direct bandgap, Urbach tail and indirect bandgap can be identified and fitted using the method described by Tran, et al. [35], as shown as dashed lines in Figure 4b. For GeSn, it has been reported that the pseudomorphic GeSn on Ge may have a direct bandgap at Sn composition of ~17%. Therefore, the Γ and L valley separation in this 16.7% Sn sample may be only a few meV. As a result, the indirect absorption is considerably weak and cannot be resolved. The direct bandgap absorption was fitted with the wavelength cut-off at ~2460 nm (dotted line). Note that the indirect bandgap energy extracted from fitting is not accurate due to the weak indirect absorption. The ellipsometry measurement in this work focuses on the estimation of direct band edge.

Figure 4c shows the PL spectra of region I at 10 and 20 K. The PL peak is observed at ~2450 nm, corresponding to the bandgap energy of 0.506 eV. The measured PL peak is in good agreement with ellipsometry measurement shown in Figure 4b. Note that according to theoretical calculation, the bandgap energy of relaxed 16.7% Sn alloy in Γ valley is between 0.32 and 0.42 eV at room temperature, depending on the selected bowing parameter. Moreover, the compressive strain will shift conduction band edge upwards while valence band downwards, and consequently will increase the bandgap energy for a few tens of meV [37]. Therefore, the measured PL peak energy of 0.506 eV at 10 K is consistent with the bandgap calculation using abovementioned method. On the other hand, the relative low intensity of PL peak at low temperature suggests that the material quality can be improved by optimizing the growth recipe in low pressure growth window. Moreover, the low peak intensity is also attributed to weak absorption with thin active GeSn layer (40 nm).

**Figure 5:**
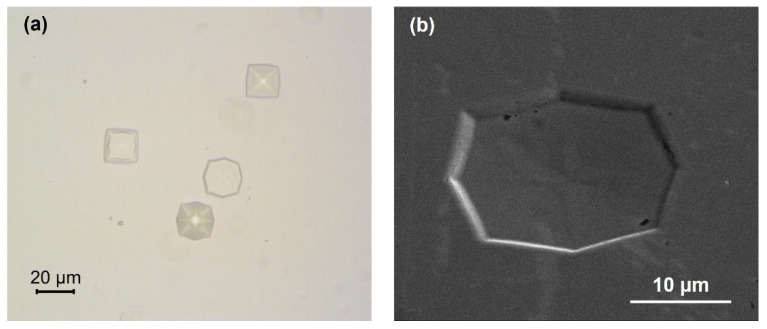
(**a**) Optical image of defects in region I. (**b**) SEM image indicating pyramidal island defects in region I.

Optical image in Figure 5a shows a few pyramidal defects in region I. To further understand the origin of the defects, the scanning electron microscope (SEM) image was taken using a FEI Nova Nanolab 200 (Hillsboro, OR, USA) to probe the details. These defects are originated from Ge buffer layer as islands during the material growth [38]. The initial islands are formed as microscale pyramidal defects, which then propagate to GeSn layer surface due to that the growth rate of Ge buffer not being low enough (0.2 µm/min) to suppress the formation of islands, resulting in 3-dimentional growth dominated by Volmer-Weber growth mechanism instead of desired Stranski-Krastanov growth mechanism [39]. The formation of pyramidal defects is believed to be caused by contamination from hydrogen clusters at the Si substrate/Ge buffer interface. These clusters hinder the mobility of Ge adatoms along the sample surface and create favorable nucleation sites in Ge layer. Similar defects were observed and reported elsewhere [31]. Since these defects would act as non-radiative recombination centers and consequently would degrade the device performance, the growth recipe needs to be optimized to reduce the defect density.

It is worth noting that during the GeSn layer growth, the breakdown of precursors begins at the edge of wafer and continues towards center. At growth temperature of 260 °C, the SnCl_4_ decomposes completely while the GeH_4_ does not fully decompose, resulting in the excess of Sn adatoms over Ge at the edge of wafer. As the precursor gases continue to contact wafer surface the ratio of Sn over Ge is reduced and the formation of Sn agglomerations is suppressed, which improves the material quality near the center of wafer. This leads to two distinct regions shown in Figure 1a, with the higher quality region at the center.

The GeSn growth pressure of 12 Torr was selected in the following way: (i) The breakdown rate of Ge precursor GeH_4_ increases as the pressure increases. To obtain the High Sn incorporation without Sn agglomeration at the surface due to Sn overabundance, the pressure of greater than 10 Torr is preferred; (ii) The Sn precursor SnCl_4_ used in our UHV-CVD system relies on the vapor pressure of the liquid in the bubbler, which limits the maximum growth pressure to be less than 20 Torr; and (iii) Based on the previous studies [34,36], reducing the SnCl_4_ partial pressure would facilitate the Sn incorporation, and therefore the Ar carrier gas was also used to control the SnCl_4_ partial pressure. The growth recipe including pressure, temperature, Ge/Sn ratio, etc. can be further optimized to improve the material quality as well as to enhance the Sn incorporation.

## 4. Conclusions

In this work, a GeSn alloy was grown using commercially available GeH_4_ and SnCl_4_ precursors via a home-built UHV-CVD reactor at low pressure of 12 Torr. The Sn composition of 16.7% was achieved as identified by XRD measurement. The optical characterizations including Raman spectroscopy, ellipsometry spectroscopy, and PL showed the shift of Ge-Ge LO phonon peak, spectral cut-off at ~2460 nm, and emission peak at ~2450 nm at 20 K, confirming the successfully grown GeSn alloy. Moreover, the analysis of pyramidal defects indicated the Volmer-Weber growth mechanism in Ge buffer. The growth results reported in this work indicate a new low-pressure GeSn growth window.

## Figures and Tables

**Table 1:** Summary of GeSn growth using SnCl_4_ via CVD reactor.

Ge Precursor	Pressure (Torr)	SnCl_4_ Flow Ratio	Temperature (°C) ^1^	Sn Incorporation	Ref.
GeH_4_	2	0.0025	250–270	2.1–8.7%	[25]
		0.005	270	6.0%	[25]
		0.01	240–350	1.5–7.9%	[25]
	40	0.0085	290–350	2.5–9.1%	[28]
		0.012	290–350	3.0–10.0%	[28]
	50	0.0006	325–335	6.0–7.0%	[27]
		0.0008	335	5.0%	[27]
	120	0.0007	280–320	8.0–18.0%	[26]
Ge_2_H_6_	N.A.	0.0045	340–400	5.0–14.0%	[30]
	45	0.01	375–475	3.5–10.0%	[29]
	100	0.02–0.04	300	9.9–10.6%	[31]
		0.027–0.05	320	5.8–7.8%	[31]
		0.0532	301–349	6.0–15.0%	[32,33]
	760	0.04	320	6.6%	[31]
**This work** ***GeH_4_***	***12***	***0.0025***	***260***	***16.7%***	

^1^ Lower temperature has the higher Sn incorporation.

## Data Availability

Data sharing is not applicable to this article.
